# Effects of early postnatal life nutritional interventions on immune-microbiome interactions in the gastrointestinal tract and implications for brain development and function

**DOI:** 10.3389/fmicb.2022.960492

**Published:** 2022-11-23

**Authors:** Jane A. Mullaney, Nicole C. Roy, Christine Halliday, Wayne Young, Eric Altermann, Marlena C. Kruger, Ryan N. Dilger, Warren C. McNabb

**Affiliations:** ^1^Riddet Institute, Massey University, Palmerston North, New Zealand; ^2^AgResearch, Palmerston North, New Zealand; ^3^High-Value Nutrition National Science Challenge, Auckland, New Zealand; ^4^Department of Human Nutrition, University of Otago, Dunedin, New Zealand; ^5^School of Food and Advanced Technology, College of Sciences, Massey University, Palmerston North, New Zealand; ^6^School of Veterinary Science, Massey University, Palmerston North, New Zealand; ^7^School of Health Sciences, College of Health, Massey University, Palmerston North, New Zealand; ^8^Department of Animal Sciences, University of Illinois at Urbana-Champaign, Urbana, IL, United States

**Keywords:** diet, gastrointestinal microbiota, gastrointestinal-brain development, immune development, early life, microbiota-GI-brain axis

## Abstract

The gastrointestinal (GI) microbiota has co-evolved with the host in an intricate relationship for mutual benefit, however, inappropriate development of this relationship can have detrimental effects. The developing GI microbiota plays a vital role during the first 1,000 days of postnatal life, during which occurs parallel development and maturation of the GI tract, immune system, and brain. Several factors such as mode of delivery, gestational age at birth, exposure to antibiotics, host genetics, and nutrition affect the establishment and resultant composition of the GI microbiota, and therefore play a role in shaping host development. Nutrition during the first 1,000 days is considered to have the most potential in shaping microbiota structure and function, influencing its interactions with the immune system in the GI tract and consequent impact on brain development. The importance of the microbiota-GI-brain (MGB) axis is also increasingly recognized for its importance in these developmental changes. This narrative review focuses on the importance of the GI microbiota and the impact of nutrition on MGB axis during the immune system and brain developmental period in early postnatal life of infants.

## Introduction

The gastrointestinal (GI) tract represents one of the largest sites of interaction between environmental factors, the host, and the microbiota (Thursby and Juge, 2017). The GI microbiota, a collection of microorganisms including bacteria, archaea, and eukarya, resides within the GI tract (mouth to anus) and has co-evolved with the host to form intricate and mutually beneficial or detrimental relationships (Laursen, 2021). Benefits include immune development through strengthening and maintaining epithelial integrity of the small and large intestine, with protective effects against pathogens through physical exclusion. The GI microbiota may also directly influence host metabolism. For example, it directly breaks down foods and food remnants as they progress through the GI tract, altering gene expression of the mucosal cells to facilitate the breakdown of foods, and producing enzymes that are not encoded in the human genome that perform digestion of food remnants (Rowland et al., 2018; Zhao et al., 2019).

There is a critical time window for microbial colonization in early postnatal life, ‘setting the scene’ for continued GI, immune, and brain development (Tooley, 2020). Disruption of the colonization process or microbiota composition (i.e., dysbiosis) can negatively affect the host, and has significant consequences for the immune system and brain development, thereby impacting cognitive function and behavior (Cussotto et al., 2018; Laursen, 2021). The bidirectional communication pathways between the GI tract and brain are termed the microbiota-gut-brain (MGB) axis; these pathways have been increasingly studied as a potential target for therapeutic applications in preventing or treating a disease or reducing disease symptoms (Brett and de Weerth, 2019). However, the mechanisms underpinning the MGB axis and their roles in the critical window of microbiota colonization during the first 1,000 days of postnatal life warrant more research.

Nutrition is among the foremost determinants of, and directly affects, GI microbiota colonization, composition, and function (Matsuyama et al., 2019). Dietary diversity is positively correlated with greater microbial diversity, due to the increased availability of metabolic substrates for the large intestinal GI microbiota (Heiman and Greenway, 2016). During the first 1,000 days, nutritional intervention is considered to have the most potential in shaping microbiota structure and function and their consequent impact on the GI, immune system, and brain development (Osadchiy et al., 2019; Tooley, 2020).

This narrative review summarizes current knowledge surrounding the importance of the microbiota for development of the GI tract, immune system, and brain during early postnatal life. The review will then discuss the impact of nutrition has on the GI microbiota and its potential role in developing these systems in early postnatal life and highlights new areas of research.

## Microbiota colonization of the gastrointestinal tract and Its effect on host–microbe interactions

While for some time there has been debate over whether the infant is born with a sterile GI tract, recent evidence supports the hypothesis that bacterial DNA are not present within the placenta and almost all microbial DNA detected accounted for by either the introduction during delivery (caesarian) or laboratory reagent contamination (de Goffau et al., 2019).

**Figure 1:**
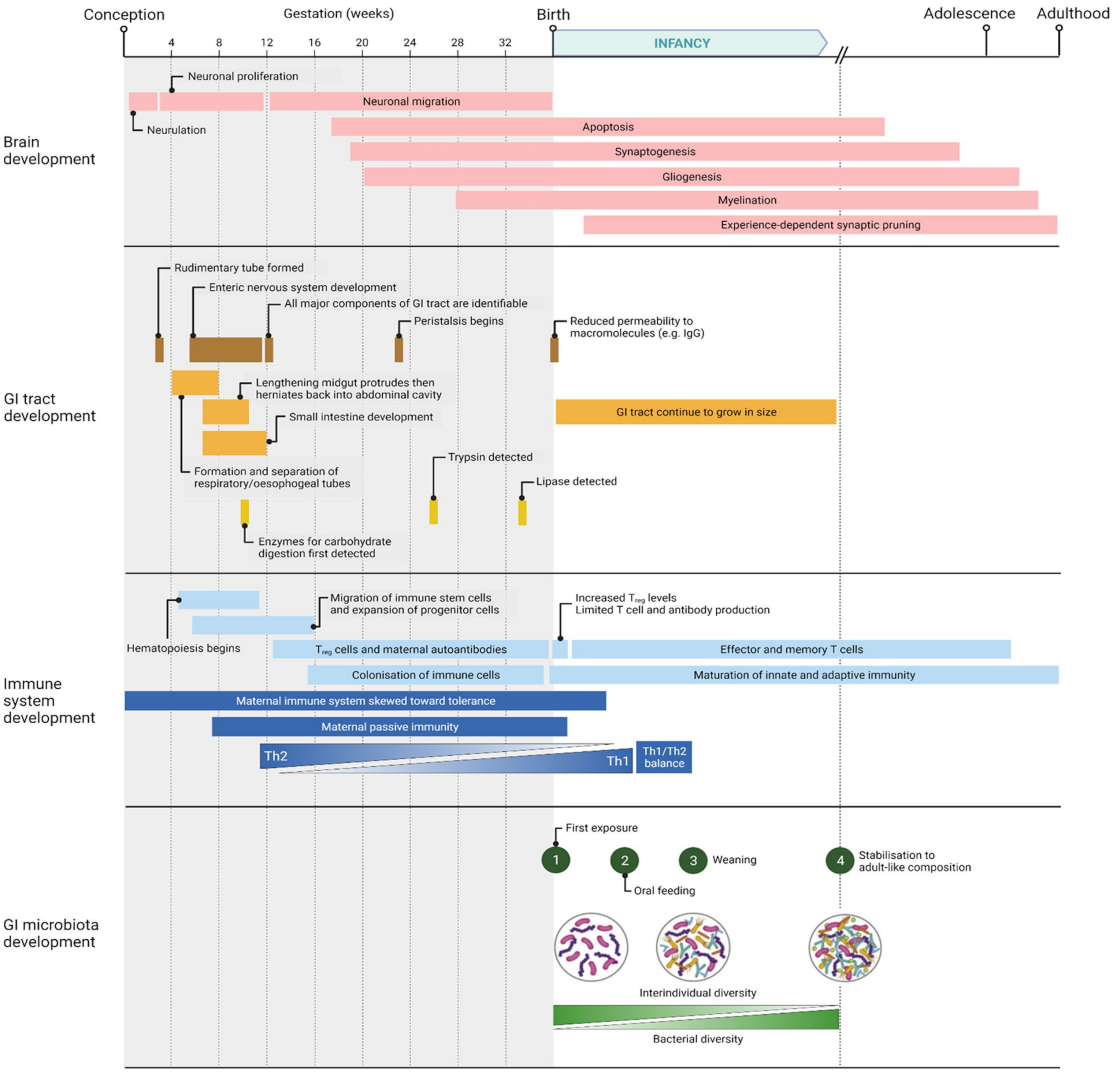
Timeline of major events occurring in brain, GI tract, immune system, and GI microbiota development from conception to adulthood. Created with BioRender.com.

Following birth, there are broadly four phases of microbial colonization of the GI tract: 1, first exposure as the infant is born; 2, continuous stimulation of the microbiota as a result of oral feeding; 3, additional stimulus of the microbiota with the introduction of solid foods (weaning transition); and 4, stabilization of the composition into an adult-like microbiota (Walker, 2017; Laursen, 2021). These phases are summarized in Figure 1, which also shows the parallel development of the infant’s GI tract, immune system, and brain. These various phases of microbiota colonization are affected by different factors: mode of delivery, infant feeding methods, gestational age, weaning duration, frequency and severity of infection, and antibiotic exposure (Rinninella et al., 2019). The composition can therefore fluctuate in response to such factors but stabilizes at around 3 years of age.

Microbial distribution and diversity also increase along the GI tract due to nutrient, chemical and oxygen availability from the proximal to the distal portions. There is a more acidic, oxygenic, and antimicrobial environment in the small intestine than in the colon, where pH is closer to neutral and the colonocytes rapidly consume oxygen (Donaldson et al., 2016). Here, the obligate anaerobes outnumber aerobic and facultative anaerobic bacteria by 100-to 1,000-fold (Donaldson et al., 2016).

Successive pregnancies or maternal parity is also a known risk factor in infant health such as growth, mortality, and even premature birth making parity a significant contributor to the microbiome during pregnancy and infant health (Berry et al., 2021). Because of this, parity should also be considered when designing microbiome studies involving pregnancy and infants.

### The microbiota-gut-brain axis

The association of the microbiota with the host, known as the MGB axis, involves bidirectional signaling pathways, and encompasses several organ systems: GI microbiota, enteric nervous system, endocrine system, immune system, central nervous system, autonomic nervous system, hypothalamic–pituitary–adrenal axis, and vagus nerve (Hyland and Cryan, 2016; Martin et al., 2018; Ceppa et al., 2019). Communication within the MGB axis is regulated using neural, endocrine, immune, and metabolic pathways (Martin et al., 2018; Osadchiy et al., 2019). It involves several neurotransmitters and metabolites (e.g., essential vitamins, secondary bile acids, amino acids, and organic acids) which concentrations must be balanced to maintain homeostasis (Petra et al., 2015; Martin et al., 2018).

Our understanding of the role that the GI microbiota plays within the MGB axis has advanced through research with animal models. While possible, clinical studies performed in humans are difficult, often limited to the collection of fecal samples as a representative of the microbiota composition of the large intestine (Romijn and Rucklidge, 2015). Non-human primates are most comparable to humans; they share physiological, metabolic, and genetic similarities (Zheng et al., 2021). However, their ethical concerns, maintenance cost, and lifespan limit their use for research (Weatheall, 2006; Cowan et al., 2020; Berding et al., 2021; Doifode et al., 2021; Margolis et al., 2021).

Fecal microbiota transplants in humans, which involves the transplant of the fecal microbiota from one individual to another, have also been used to study the MGB axis (Codagnone et al., 2019; Cryan et al., 2019). When successful, the recipient’s microbiota compositional signature or profile is more like the donor than the recipient (Cryan et al., 2019). It has been observed in several studies that behavioral phenotypes, for example depression or anxiety, may be transferred along with the microbiota (Bercik et al., 2011; Bruce-Keller et al., 2015; Kelly et al., 2016; Zheng et al., 2016). Infants may benefit as well. In a proof-of-concept study, the transfer of maternal fecal microbiota was shown to restore normal microbiota development in cesarean-born infants (Korpela et al., 2020).

Of research involving humans, it has been reported that the MGB axis involved in infant development connects general neurocognitive development, socio-emotional behavior, and physical brain structure where function is assumed from neuroimaging (Vaher, 2022). Substantial variation exists across studies, however, most likely due to the methodologies, sample sizes, DNA sequencing methods, and even the statistical models applied. Standardization of sequencing methods and carrying out whole metagenome analyses are required to improve our understanding of the role of the gut microbiome in brain development during those early postnatal development stages (Vaher, 2022). Further research is needed in infants using methods to assess the role of the MGB axis in longitudinal brain development and behavior.

Researchers have applied other methods of studying the MGB axis in mice which includes the use of antibiotics to alter the microbiota composition to elicit behavioral or cognitive changes (Douglas-Escobar et al., 2013; Hoeman et al., 2013; Chu et al., 2019). In another recent study, changes in the microbiota of adult mice brought about with antibiotics or germ-free conditions resulted in behavioral changes, specifically, deficits in fear extinction learning (Chu et al., 2019). This behavior deficit could be restored through by reestablishment of the microbiota suggesting that the microbiota produce compounds that are able to directly affect brain function. It may be also that probiotics can prevent brain injury through blocking the ability of damaging molecules to reach the brain *via* the MGB axis (Douglas-Escobar et al., 2013). Immune systems work in concert. For example, neonates also develop maturation of their dendritic cells, which is brought about through exposure to the microbiota. The dendritic cells produce interleukin (IL)-12 which accumulates in the environment and through a mechanism where IL-12 offsets the bias toward Th1, thereby promoting Th1 differentiation to Th2 (Hoeman et al., 2013).

Pigs are a more suitable and preferable choice over rodents for research focusing on early postnatal life development of the human infant. Pigs and human infants share similar scaled GI and brain developmental timelines as compared to rodents (Mudd and Dilger, 2017). In general, the porcine brain is one-tenth the total volume of the human brain throughout its lifespan with 1 month of human growth being approximately equivalent to 1 week of piglet total brain growth. This is in contrast to rodent brains, which are much smaller, grow and develop with a different trajectory, and would often require pooling tissue to analyze its components (Mudd and Dilger, 2017). Furthermore, the precocial nature of piglets allows for behavioral and cognitive assessments to be performed immediately after birth, which is impossible in either rodents or humans (Mudd and Dilger, 2017; Val-Laillet, 2019). Additionally, most knowledge in immune ontogeny in early postnatal life has stemmed from studies with germ-free rodents, which provide results that are not readily translatable to humans. Pigs are, again, an improved animal model as they share greater similarities with humans in terms of anatomy and function of the immune system (e.g., pigs possess tonsils whereas rodents do not; Pabst, 2020).

Pigs are also the animal model of choice in terms of digestive and associated metabolic processes, as their nutritional requirements when compared with other non-primate animal models are more closely aligned to those of human (infants; Kobek-Kjeldager et al., 2022). Cannulation is also possible for repeated, stress-free digesta sample collection to assess nutrient digestibility and kinetics over the lifetime (Bennell and Husband, 1981a,b). Therefore, the use of pigs in early postnatal life developmental research allows for the determination of the critical time windows of GI, brain, and immune development that may be sensitive to nutrition intervention.

### Development of the immune system

Newborn infants need time to develop and mature their innate and adaptive immune systems. There are some transfers of maternal immunity both *in utero* and through breast milk. These processes help to provide immunity to the infant in the first days through transfer of both secretory immunoglobulins and ‘milk leukocytes’, which produce molecules that migrate to the GI and respiratory tracts (Tuaillon et al., 2009; Houghteling and Walker, 2015; Rudzki et al., 2017). Over time, the components of breast milk change from a primarily protective to a nutritional role (O’Neill et al., 2020; Laursen, 2021).

The maturation of the immune system requires the GI microbiota, the absence of which impacts all aspects of immune system development and function (Mohajeri et al., 2018). Improper development brings about several immunological defects, including increased susceptibility to infections and altered immune homeostasis (Cong et al., 2015; Nash et al., 2017; Eshraghi et al., 2018). It has been observed that mice treated with antibiotics, or gnotobiotic mice colonized by a limited defined consortia of bacteria, showed impaired microglia maturation and immune response upon bacterial stimuli when compared with their conventional counterparts (Erny et al., 2015). Training of the infant immune system is also required for optimal GI function, including vascular supply, epithelial healing, nutrient absorption, and protection from infection.

There is an interface between innate and adaptive immunity, where T or B cells are recruited and work at protecting intestinal mucosal from the resident microbes. The immune system includes the Toll-like receptors (TLRs) that recognize the presence of microbes from their DNA to their surface molecules to the specialized T cells. For a review of ‘the interface between innate and adaptive immunity’ see Hoebe et al. (2004).

Innate immunity includes the mucosal-associated invariant T (MAIT) cells, abundant in humans and a key immune system component. MAIT cells are atypical T-cells with a limited response repertoire activated by riboflavin-derived molecules (rather than peptides), which are presented through the major histocompatibility complex (MHC) class I protein MR1 (Godfrey et al., 2019). MAIT cells are absent in germ free mice and become more abundant in microbial-challenged mice, so it is highly likely that exposure to and association with microbes in the GI tract is responsible for their development and maturation (Chen Z. et al., 2017; Godfrey et al., 2019). Similarly, invariant natural killer (NK) T cells are abundant in healthy infants and have a role as ‘cytotoxic effectors’ and regulation of the adaptive immune system (Wingender et al., 2012; Sagebiel et al., 2019).

Immunoglobulins are also part of the innate and adaptive immune response and are critical to the infant’s ability to specifically recognize and bind to antigens, which facilitates their destruction. There are five main classes of immunoglobulins (IgG, IgM, IgA, IgD, and IgE), characterized by the type of heavy chain within their structure, resulting in differences in their function and type of immune response elicited by each molecule. Secretory IgA (sIgA) is transferred through maternal breast milk to the infant where it protects from infection and is critical for homeostasis of the microbiota not only through encouraging colonization but through influencing the microbiota gene expression (Mirpuri et al., 2014; Rogier et al., 2014; Gopalakrishna et al., 2019; Zheng et al., 2020). Maturation of IgA and IgG requires B cell class-switching, and this process does not mature until around 6 months of age. IgE may also be produced through B cell isotype switching at mucosal sites and abnormally high plasma IgE levels have also been observed in germ-free mice compared with conventionally reared mice. A study on this concluded that a sufficient microbial stimulation during early postnatal life is required to maintain baseline IgE levels (Cahenzli et al., 2013). Additionally, it has been demonstrated that the ‘allergy phenotype’ is transferrable *via* transplantation of the GI microbiota (Walker, 2017; Smeekens et al., 2020). Germ-free mice are inherently susceptible to anaphylactic responses to food, quantified by a drop in body temperature. The colonization of these mice with the microbiota from healthy infants protected the mice from anaphylactic responses, but not when colonized using the microbiota from infants suffering from bovine milk allergy (; Smeekens et al., 2020).

Research in rodents have shown that appropriate innate immune responses are also required for nutrient absorption and metabolism. Shulzhenko et al. identified inter-connecting regulatory signaling networks which balance metabolism and the innate defensive mechanisms in epithelial cells (Shulzhenko et al., 2011). If IgA concentrations are altered, these networks become unbalanced and may cause irregular upregulation of certain pathways (e.g., innate immunity), while downregulating others (e.g., lipid uptake and metabolism; Shulzhenko et al., 2011). Furthermore, bacterial fermentation of indigestible dietary fibers produces organic acids in the colon (Silva et al., 2020). Organic acids promote intestinal barrier integrity, mucus production, and supporting a tolerogenic response over inflammation (O'Keefe, 2016; Parada Venegas et al., 2019; Silva et al., 2020; Laursen, 2021).

Adaptive immunity involves the maturation of T and B cells which begin their development while still *in utero* (Haynes et al., 1988; Haynes and Heinly, 1995; Schonland et al., 2003; Rechavi et al., 2015). Infant responses to antigen by T and B cells are known to be weaker than in adults in most cases except in some vaccines and pathogens (Siegrist and Aspinall, 2009). It is important to acknowledge that much of our understanding early postnatal life immunity in infants has relied on murine models even though they differ substantially in ontogeny as reviewed by Semmes et al. (2020).

### Role of the gastrointestinal microbiota in brain development

Brain development begins *in utero* and continues into adolescence (Zuckerkandl and Pauling, 1965), as shown in Figure 1. Major events of brain development in early postnatal life include synaptogenesis, myelination, and synaptic pruning. The GI microbiota is known to affect or be associated with these events, as well as influence neural development, cognition, and behavior. Cognitive functions, including learning capacity and memory, are closely linked with the GI microbiota (Liang et al., 2018b). Cognitive function encompasses the life-long process of learning, which includes both long-and short-term processes (Gareau, 2016). Cognitive impairment has been noted in individuals with GI or neurological disorders (Gareau, 2016; Vuong et al., 2017). Several human studies have been published already, correlative, linking the microbiome with brain function and a summary of these and the findings has been reviewed recently (Tooley, 2020). One recent study looked at the fecal microbiota profiles of 1-year-old infants and correlated these with regional brain volume data and Mullen Scales of Early Learning (indicator of cognitive performance) at 2 years of age, finding differences (Carlson et al., 2018). Although brain volume was not different between groups, the fecal microbiota alpha diversity differed between age groups (Carlson et al., 2018). A key finding in that study was that highest level of cognitive performance could be predicted by the *Bacteroides* genus while the lowest level of performance was predicted by the *Faecalibacterium* genus. While there was a difference in cognitive performance depending on microbiome diversity, there was no evidence of a ‘cognitive damage’ microbiota profile or nor any cognitive impairment in either age group (Carlson et al., 2018).

Studies using germ-free animal models or antibiotic-induced dysbiosis have been used to demonstrate that without a ‘normal’ GI microbiota, working and spatial memory are negative influenced (Manderino et al., 2017; Vuong et al., 2017). For instance, elevated hippocampal levels of serotonin (Jameson and Hsiao, 2018) and brain-derived neurotrophic factor (Borrelli et al., 2016; Hoban et al., 2016) were linked with behavioral changes between germ-free and conventional rodents, such as increased depressive-like and decreased anxiety-related behaviors (Cryan and Dinan, 2012; Foster and McVey Neufeld, 2013; Guida et al., 2018; Fulling et al., 2019). The decreased anxiety-like behavior in germ-free mice was observed when subjecting the mice to various tests that measure the natural aversion of rodents for open and elevated areas and their natural, spontaneous exploratory behavior in novel environments. This cautious versus exploratory behavior must be balanced to ensure survival of the individual for procreation. While decreased anxiety may be advantageous, this may lead to an increased chance of predation and therefore decrease their likelihood of survival. The exhibited aberrant behavior persisted following the colonization of these germ-free mice with a conventional microbiota. These findings show that the GI microbiota plays a role in developing stress pathways and a critical time window exists for reconstitution of the microbiota to normalize behavior (Cryan and Dinan, 2012; Foster and McVey Neufeld, 2013; Guida et al., 2018; Fulling et al., 2019). Similar findings have been reported where a critical time window of colonization exists to avoid negative behavioral changes in adulthood (Sudo et al., 2004; Diaz Heijtz et al., 2011). Furthermore, a study by Bercik et al. showed that behavioral changes are transferable following transplantation of the GI microbiota (Bercik et al., 2011).

The GI microbiota is also linked to changes in neurogenesis (Gareau, 2016; Liang et al., 2017, 2018a; Wei George et al., 2021). In a recent review on how the microbiota composition impacts neurogenesis, possible strategies for using the microbiome to treat neurological disorders is discussed but the mechanisms for microbiome inhibition or promotion of neurogenesis are still not understood (Liu et al., 2022). The primary mode of communication from the GI microbiota to the host’s central nervous system is achieved through immune or endocrine mechanisms (Martin et al., 2018). These mechanisms are often mediated by microbially-derived molecules such as organic acids and tryptophan metabolites (Wikoff et al., 2009; Tolhurst et al., 2012; Yano et al., 2015). For example, some 90% of serotonin required for mood, behavior, sleep, and several other functions within the central nervous system and GI tract is produced by the intestinal microbiota (Gershon and Tack, 2007; Gershon, 2013). These metabolites signal enteroendocrine and enterochromaffin cells, which in turn may act directly or indirectly on the central nervous system (Martin et al., 2018). Direct signaling must overcome obstacles such as the epithelial barrier and immune system in the GI tract or blood–brain barrier to exert its effects on the brain (Samuel et al., 2008; Haghikia et al., 2015; Yano et al., 2015). Indirect signaling may induce responses in the central nervous system through long-distance neural signaling by vagal and/or spinal afferents (Goehler et al., 2005; Bravo et al., 2011). Alterations to these signaling pathways within the MGB axis have been implicated in the pathogenesis and pathophysiology of both functional GI and neurological disorders (Rhee et al., 2009; Mayer, 2011). An approach may be developed for prevention and treatment by targeting specific mechanisms through which the GI microbiota interacts and contributes to these disorders (Rhee et al., 2009; Mayer, 2011; Mayer et al., 2014; Cerdó et al., 2017).

Organic acids produced primarily by the colonic microbiota have been observed to exert effects on blood–brain barrier permeability (Braniste et al., 2014). The three most abundant organic acids are the short-chain fatty acids acetate, propionate and butyrate (Rios-Covian et al., 2016). Acetate produced in the intestine crosses the blood brain barrier and is taken up by the brain where it is incorporated into the hypothalamus and eventually has a role in appetite regulation (Frost et al., 2014). Propionate has an effect on the regulation of the sympathetic nervous system, and both propionate and butyrate affect intracellular potassium levels (Oleskin and Shenderov, 2016), and also regulate the expression of tryptophan hydroxylase, which in turn is involved in the biosynthesis of serotonin (Nankova et al., 2014). For a review on the role of the organic acids in the MGB axis see Silva et al. (2020).

The GI microbiota plays a role in the normal development and regulation of the hypothalamic–pituitary–adrenal axis. This axis is a major component of the homeostatic response that mediates the effects of stressors by regulating many physiological processes. Thus, the GI microbiota can influence the host’s stress reactivity and anxiety-like behaviors (Saulnier et al., 2013; de Weerth, 2017). For example, maternal separation stress models, e.g., where young are separated from their mothers to stimulate a stress response, have been used to assess how acute and/or chronic stress affects the mouse pups. Stress has resulted in memory dysfunction in germ-free rodents, attributed to altered brain-derived neurotrophic factor expression levels (Gareau et al., 2011). This factor regulates several aspects of the brain, and its altered expression can lead to downstream effects on cognitive functions and intestinal muscle repair, regeneration, and differentiation (Al-Qudah et al., 2014). Germ-free animals also exhibited decreased anxiety and increased stress response with augmented levels of adrenocorticotropic hormone and cortisol (Diaz Heijtz et al., 2011; Neufeld K. A. et al., 2011; Neufeld K. M. et al., 2011; Clarke et al., 2013; Nishino et al., 2013). Following recolonization of the GI tract with a conventional microbiota, normalizing the hypothalamic–pituitary–adrenal axis occurs in an age-dependent manner (Sudo et al., 2004). Reversibility of the increased stress response is achievable only in germ-free mice aged less than 3 weeks, supporting the notion of a critical period during which the plasticity of neural regulation is sensitive to input from the GI microbiota (Sudo et al., 2004).

Stress during early postnatal life has been shown to have long-lasting effects, including altering the MGB axis. A study by O’Mahoney et al., using the maternal separation rat model, showed altered composition of the fecal microbiota following early postnatal life stress as well as altered behavior and systemic immune responses compared to a control group (O'Mahony et al., 2009, 2011). Furthermore, basal adrenocorticotropic hormone levels and increased anxiety-like behaviors were higher in the stressed groups when compared with the control group (Daniels et al., 2004). However, following a subsequent stressor, adrenocorticotropic hormone levels were lower in the stressed groups and were accompanied by altered neurotransmitter levels, indicating that the stressor had detrimental effects on regular stress responses and induced abnormal behaviors (Daniels et al., 2004; Saulnier et al., 2013; Rincel and Darnaudéry, 2019). Psychological stress can directly affect the integrity of the tight junction proteins responsible for barrier integrity for the intestine and the blood brain barrier and loss of this integrity is also correlated with microbiota dysbiosis (Geng et al., 2019). Chronic stress can also create a loop that affects memory through a feed-forward loop mechanism leading to depressive disorders as shown in Japanese quails (Kraimi et al., 2022). In this animal model study, Japanese quails experienced induced stress with their microbiota transferred to unstressed quails. Again, the *Bacteroides* genus was implicated with the *Alistipes* genus showing increased abundance in the stressed group and this stress response was transferred through the microbiota to the unstressed recipient (Daniels et al., 2004; Saulnier et al., 2013; Rincel and Darnaudéry, 2019; Kraimi et al., 2022).

## Early postnatal life nutrition and the microbiota-gastrointestinal-brain axis

The microbiota is susceptible to modulation by external factors prior to stabilization of its composition at approximately 2–3 years of age. Among these, infant diet has been identified as a major contributor to GI microbiota development in early postnatal life. As such, the effects of infancy diet (namely formula vs. breast milk) on early postnatal life development have been well documented (Colen and Ramey, 2014; Kowalewska-Kantecka, 2016). Comparably, less is known about the effects complementary feeding has on infant microbiota composition (Laursen et al., 2017; Laursen, 2021). Given the dominating influence diet and nutrition has on microbiota composition, and the involvement of the microbiota in regulating immune and brain development, gaining a deeper understanding of the potential microbe-mediated host effects of feeding mode in early infancy is needed (Conlon and Bird, 2014). Also, the complementary feeding period (6–24 months of life) coincides with a critical period in microbiota development, transitioning away from the influence of a milk-based diet (Laursen et al., 2017; Laursen, 2021). The infant microbiota composition stabilizes and resembles an adult-like microbiota at around 3 years of age and attempts at modulation are likely to be more successful if they are conducted before this or early in this period to elicit any beneficial downstream effects.

### Early infancy diet

Breast milk is the recommended first nutrition for the infant, providing all necessary nutrients to support growth and development, as well as passive immunity to protect against infectious diseases during infancy. After lactose and lipids, human milk oligosaccharides (HMO) are the third most abundant component of breast milk. These comprise short saccharides composed of five monomeric building blocks (glucose, galactose, fucose, N-acetylglucosamine, and sialic acid), of which over 200 different structures have been identified. These oligosaccharides are responsible for selectively promoting the growth and function of beneficial bacteria As infants lack the necessary enzymes to digest HMOs, the molecules pass into the large intestine and function as a carbon source for commensal bacteria (Yang et al., 2016; Walker, 2017), promoting and stimulating the growth of specific bacterial groups such as *Staphylococci* (Hunt et al., 2011), and from genera *Bifidobacterium* (Zivkovic et al., 2011), *Streptococcus*, *Lactobacillus* (Harmsen et al., 2000) and *Bacteroides*. Only bacteria such as *Bifidobacterium longum* subspecies *infantis* lineage harbor genes to express all enzymes required for degrading and utilizing HMOs (Milani et al., 2017; Taft et al., 2018) However, other bacteria may cleave and utilize specific components of HMOs (Milani et al., 2017). HMOs have been attributed to the two-fold increase of *Bifidobacterium* cells in breastfed infants compared to formula-fed infants (Rinninella et al., 2019). Some bacteria, including *Bacteroides fragilis*, *Bifidobacteria infantis*, and *Lactobacillus acidophilus*, stimulate endogenous production of sIgA, activation of regulatory T cells and anti-inflammatory molecules – all necessary for host homeostasis (Newburg and Walker, 2007; Chichlowski et al., 2012; Jost et al., 2012). Lactoferrin, another component in breast milk, also encourages the proliferation of beneficial bacteria such as *Lactobacillus* and *Bifidobacterium* genera (Mastromarino et al., 2014).

Breastmilk has also been demonstrated to provide passive protection and stimulate the development of the infant’s immune system (Schwartz et al., 2012). For example, polymeric IgA and defensins can interfere with pathogen attachment and uptake (Newburg and Walker, 2007), while *n-3* fatty acids (Wijendran et al., 2015) and transforming growth factor-β (Rautava and Walker, 2009) can activate enterocytes to produce anti-inflammatory cytokines, and lactoferrin can interact with the GI tract and promote immune homeostasis (Newburg and Walker, 2007; Walker and Iyengar, 2015).

Comparison of the intestinal microbiota in formula and breast-fed infants showed that at around 40 days, those fed exclusively with formula had greater alpha diversity while both breast-fed and formula-fed infants were colonized predominantly with *Bifidobacterium* species and members of *Enterobacteriaceae* family (Ma J. et al., 2020). While the diversity in the breast-fed infants was lower than formula-fed at day 40, it increased by 4 months and diversity was similar between formula and breast-fed infants. Lower diversity in breast-fed infants was most likely due to the breast milk which requires specific bacteria capable of degrading the oligosaccharides that are in the milk. Although many infant formula products are supplemented with prebiotics such as fructo-oligosaccharides and/or galacto-oligosaccharides, these are not as selective as HMOs since they can be utilized by most *Bifidobacterium* species (Akiyama et al., 2015) and stimulate the growth of various *Lactobacillus*, *Streptococcus*, and *Bacteroides* species (Schwab and Gänzle, 2011; Ma C. et al., 2020). Comparison of metabolic profiles of infants fed either exclusively formula or breastmilk have also confirmed that the metabolic capabilities of the microbiota are primarily proteolytic and saccharolytic, respectively (Chow et al., 2014; He et al., 2019).

### Weaning and complementary feeding

Over the course of infancy, a point is reached where milk-based feeding is no longer adequate to cover the nutritional requirements of the infant. Therefore, supplementation with additional foods is required. The introduction of solid foods and the progressive reduction of milk-feeding lead to major GI microbiota compositional and functional changes. Bacteria belonging to families *Bifidobacteriaceae* and *Enterobacteriaceae* are decreased after weaning (Fallani et al., 2011a,b), and any compositional differences between breastfed or formula-fed infants slowly decrease (Yaron et al., 2013; Mastromarino et al., 2014; Rinninella et al., 2019).

Pigs also produce milk oligosaccharides (pMOs); Twenty-nine were described in 2010 and were found to be abundantly sialylated making them more similar to bovine than human (Tao et al., 2010). However, some pMOS are fucosylated, are much more abundant in pigs than in bovine (9% versus 1%) which suggests that pMOs are actually more closely like human than bovine milk and therefore are also influencing the microbiota (Salcedo et al., 2016). In a neonatal piglet model, the fecal microbiota composition stabilized 10 days post-weaning (Chen L. et al., 2017). The sudden change from high-fat, low-carbohydrate milk (pre-weaning) to a high-carbohydrate, low-fat feed (weaning and onward) resulted in a drastic change of available nutrients to the commensal bacteria (Guevarra et al., 2019). The predominant genera post-weaning are microbes efficient in degrading dietary fibers and producing organic acids (Chen L. et al., 2017), resulting in a microbiota composition more adult-like (Chen L. et al., 2017; Guevarra et al., 2018, 2019). Chen et al. observed a shift from a high prevalence of *Lactobacillus* and *Bacteroides* genera, to *Roseburia, Paraprevotella* and *Blautia* genera, post-weaning (Chen L. et al., 2017). Furthermore, Firmicutes and Bacteroidetes remained the most abundant phyla pre-and post-weaning (Alain et al., 2014; Hu et al., 2016; Chen L. et al., 2017). Another study supported these findings, reporting *Bacteroides* as the most abundant genus in nursing pigs (pre-weaning), with *Prevotella* and *Lactobacillus* genera enriched in weaned pigs (Guevarra et al., 2018, 2019).

Interventions with probiotics to improve or maintain good health in early postnatal life have gained much popularity in recent decades (Kapourchali and Cresci, 2020). Some infant formula has been designed to include probiotics, for example *Bifidobacterium* and *Lactobacillus* species, to mimic the composition of human breastmilk. However, probiotic-supplemented infant formula contains a much higher concentration of these probiotic strains when compared with breastmilk (Fernández et al., 2018). Furthermore, not all probiotics are functionally equal, as the effects obtained from one strain cannot be assumed to be replicable with another strain, even if they belong to the same species (McFarland et al., 2018). Even if they are not equal, probiotics have been demonstrated to be safe and effective across multiple studies involving infants. For example, supplementation with probiotics can increase the microbial metabolism of milk oligosaccharides in infants while also reducing intestinal inflammation (Larke et al., 2022). In another recent study, probiotics demonstrated efficacy against the development of antibiotic resistance in preterm infants (Guitor et al., 2022) and in another, probiotics were shown to cause no adverse effects in vulnerable premature infants and decrease the risk of necrotizing enterocolitis (Underwood et al., 2020).

Cognitive impairment is sometimes alleviated through probiotic administration (Liang et al., 2015; Ohsawa et al., 2015; Wang et al., 2015). For example, administration of *Lactobacillus helveticus* improved the stress response and cognitive dysfunction induced by chronic stress in rats (Liang et al., 2015), and *Bifidobacterium longum* strains were observed to be effective in improving memory (Ohland et al., 2013; Savignac et al., 2015). Early postnatal life probiotic intervention has also been observed to reduce sepsis and allergy, as well as having a possible role in reducing the risk of neuropsychiatric disorders such as autism spectrum disorder and attention deficit disorder (Weizman et al., 2005; Indrio et al., 2014; Pärtty et al., 2015; Korpela et al., 2016; Panigrahi et al., 2017). There is also evidence that a combination of *Streptococcus thermophiles* and *Bifidobacterium* genus can be effective in preventing the onset of diarrhea in children following antibiotic treatment (Corrêa et al., 2005). Four *Lactobacillus* strains isolated from breastmilk protected against infection in a mouse model, with potent antimicrobial properties (Olivares et al., 2006). Thus, cultivating microbes from human breastmilk may also prove to provide good probiotic candidates for further research and development.

The application of prebiotics and probiotics in improving health in early postnatal life is promising, but the timing of administration, the quantity administrated, the effect of different strains, combination of strains, engineering, and safety must be carefully considered and continually researched to fully understand how they modulate the GI microbiota composition and exert their effects.

There is a developmental aspect of the growing infant where multiple maturation events are proceeding in the brain; in one direction is synaptogenesis, microglia maturation, with targeted synaptic pruning and myelination. Meanwhile, in the immune system, T and B cells are maturing, innate immune cells are being trained through exposure to the environment, and the immune system is learning to differentiate self from non-self (i.e., foreign). Similarly, in parallel, the microbiota in the GI tract is also maturing, colonizing the intestine, metabolizing nutrients, and releasing microbial products that may be immune modulatory or in the case of butyrate, an energy source for enterocytes lining the intestinal epithelium. The vagus nerve sits right at that junction of the MGB axis and is an integral part of the communication between systems. It accesses the entire digestive wall yet does not cross the epithelial layer and it senses and responds to the signals that come from the microbiota transported across the epithelial layers by host entereroendocrine cells (Wang and Powley, 2007). If this communication system the vagus nerve was targeted, it may be able to affect and restore the homeostasis of the MGB axis (Bonaz et al., 2018).

## Future perspectives

The mutualistic relationship that exists between the host and microbes begins at birth and shapes both host health and microbiota composition and function. The development and maturation of the GI tract, immune system, brain, and microbiota are in turn influenced by host genetics and exposure to the environment (e.g., diet, delivery mode, feeding methods, weaning, infection, and antibiotics). There are important interactions between the development and maturation of the immune system that drive the establishment and maturation of the microbiota in the GI tract and potentially affect the brain development and function and associated cognitive behaviors in infants.

The implications of the microbiota and immunological findings discussed in this review for pregnant women, mothers, infants, infant nutrition policy makers, formula manufacturers, and health-conscious consumers are important aspects to consider. The effects of the microbiota on the mental and cognitive state of the infant cannot be ignored. Development and maturation are staged and interdependent processes, with a narrow window of opportunity where nutrition can modulate the microbiota for beneficial effects to be conferred to the infant. Appropriate nutrition might encourage more beneficial bacteria within the microbiota to flourish however, such intervention is a balance between benefits and risks.

Current evidence suggests that the MGB axis is a highway of communication and connections between two complex systems found in the host and the GI tract microbiota. The communication and interactions are complex and manipulating one system might also have unintentional negative outcomes for the other system. For example, high alpha diversity of the microbiota is recognized as an indicator of health. However, in early postnatal life, when the diet is primarily milk based, this diversity is low. The *Bifidobacterium* genus and *Enterobacteriaceae* family are the dominant in the microbiota at that age and these bacteria are critical to the immune development of the infant. The brain also relies on the appropriate immune development to develop.

Another possibility is to encourage the prevalence of fiber-degrading microbes in the infant microbiota by offering fiber-rich foods, but only after the infant transitions to solid foods and thereby has a sufficiently mature microbiota composition. Fiber-rich complementary foods then lead to the increased production of beneficial organic acids which impact positively the colonic epithelium where they are absorbed with specific organic acids (e.g., acetate) affection brain and cognition outcomes. The reverse is inflammatory conditions which can allow translocation of bacteria and their products to the blood where the downstream effects are more inflammation, oxidative stress and may lead to disease.

Increasing the abundance of the *Bacteroides* genus and reducing the abundance of the *Faecalibacterium genus* are counterintuitive to the beneficial effects of the *Faecalibacterium genus* on health. It is known that the methods of assessing the microbiota composition and function lack resolution to characterize which species or bacterial strains are involved. Consequently, some important changes in the microbiota composition that contribute to improve cognition in infants might be undetected in the preweaning period. Sequencing depth and resolution needs to increase to discriminate between bacterial species to better understand these relationships.

Appropriate nutrition in early postnatal life feeds the microbiota in the GI tract sets the baseline for immune, physical and brain health in later life. The microbiota shapes the immune system and is in turn shaped by the immune system. Interactions with any one of these systems impacts on the others. The ability to measure and assess such a dynamic set of systems will involve a cross disciplinary translational approach. It is possible that to reverse or at least mitigate the effects of inadequate nutrition through dietary interventions at that point in time, that narrow window of opportunity before the immune system, the brain and the microbiota mature into the stable adult shape which persists.

## Author contributions

CH, NR, JM, WY, EA, MK, RD, and WM have contributed to the work. CH conceived and wrote the initial draft of the manuscript. JM and NR edited and revised the manuscript and in structuring the paper and critically reviewing the manuscript. JM, NR, CH, WY, EA, MK, RD, and WM advised and critically reviewed versions of the paper. All authors contributed to the article and approved the submitted version.

## Funding

CH was supported by a fellowship from the Riddet Institute, through funding provided by the NZ Ministry of Business, Innovation & Employment Smarter Lives project. The same grant also supports co-authors JM, WY, EA, and WM.

## Conflict of interest

The authors declare that the research was conducted in the absence of any commercial or financial relationships that could be construed as a potential conflict of interest.

## Publisher’s note

All claims expressed in this article are solely those of the authors and do not necessarily represent those of their affiliated organizations, or those of the publisher, the editors and the reviewers. Any product that may be evaluated in this article, or claim that may be made by its manufacturer, is not guaranteed or endorsed by the publisher.

## Abbreviations

GI: Gastrointestinal
HMO: Human Milk Oligosaccharides
MAIT: Mucosal-associated invariant T cell
MHC: Major Histocompatibility Complex
MGB: Microbiota-GI-brain
NK cell: Natural Killer cell
Ig: Immunoglobulin
